# The French multicentric molecular analysis platforms and personalized medicine trials MOST, MOST Plus and MEGAMOST

**DOI:** 10.2340/1651-226X.2024.32745

**Published:** 2024-05-28

**Authors:** Loic Verlingue, Marine Desevre, Marie Polito, Gwenaële Garin, Christine Rodriguez, Wang Qing, Olivier Tredan, David Perol, Isabelle Ray-Coquard, Sylvie Chabaud, Jean Yves Blay

**Affiliations:** Centre Léon Bérard, Centre de Recherche en Cancérologie de Lyon, Lyon, France

**Keywords:** Personalized oncology, molecular analysis, oncology, sorafenib, olaparib

## Abstract

**Background and purpose:**

In this manuscript we describe the academic French multicentric molecular analysis platforms including PROFILER, promoted by Centre Léon Berard, and the multicentric personalized medicine trials MOST, MOST Plus and MEGAMOST.

**Patients/material and methods:**

MOST, MOST Plus and MEGAMOST comprise 14 cohorts with different targeted agents and immunotherapies.

**Results and interpretation:**

PROFILER has recruited 5,991 patients in 10 years, MOST and MOST Plus 875 patients since 2014 and MEGAMOST 172 patients since 2020, and are still ongoing. We provide a description of the local, national and international implications of these initiatives, and we review the results of the sorafenib and olaparib cohorts.

## Introduction

The first generation of personalized medicine umbrella or basket trials in France allowed 10–20% of patients to receive a targeted treatment based on molecular analysis (mainly targeted sequencing and comparative genomic hybridization (CGH)) [1–3]. It is estimated that 40–50% of patients would benefit from theoretical orientation if more treatments were available and accessible [4, 5]. Drug Rediscovery Protocol (DRUP)-like trials have the potential to increase the number of compounds and molecular markers to orient patients to targeted treatments. Centre Léon Bérard (CLB) and partnering sites have developed an environment combining multiple types of molecular analysis and orientation to academic DRUP-like trials called MOST and MEGAMOST for patients with advanced or metastatic cancers.

## Molecular analysis and sequencing programs

Several molecular analysis programs are currently running in our hospital resulting in multiple levels of molecular information (Table 1). Most of the platforms are multicentric, either regional in Rhône Alpes and centralised at CLB as for ProfiLER01, or national and centralised in dedicated sites (including CLB), as for FMG2025.

**Table 1:** Molecular screening programs at Centre Léon Bérard.

Program	Type of data	Organisation	Tools used for interpretation
ProfiLER	Tumour target DNAseq, RNAseq, CGH	Rhône Alpes: Data, analysis & MTB centralized at CLB	In house + Open source
FMG2025	WGS, RNAseq	France, reports at CLB for MTB	National
PRISMportal	ctDNAseq	France. In Rhône Alpes: Data, analysis & MTB centralized at CLB	Foundation medicine
PLANET	ctDNAseq, tumor WES, RNAseq	CLB, sequential analysis	In house

ctDNAseq: circulating DNA sequencing; WES: Whole Exome Sequencing; DNAseq: DNA Seqencing; RNAseq: RNA Sequencing; CGH: Comparative Genomic Hybridisation; MTB: molecular tumour board; CLB: Centre Leon Berard; CGI: Cancer Genome Interpreter.

### ProfiLER screening programs

The ProfiLER01 (NCT01774409) is a multicentric, prospective and non-randomised ongoing program. ProfiLER is dedicated to adult patients with advanced/metastatic cancer who progressed after at least one line of standard treatment. The current molecular analysis includes the identification of single nucleotide variants (which evolved across three different panels over time), copy number alterations (using CGH array), tumour mutational burden, microsatellite status (both implemented since 2023) and oncogenic fusion using in-house genomic workflows. ProfilER02 (NCT03163732) included FoundationOne^®^ CDX panel of 324 genes (under review). This is a multidisciplinary effort including the molecular biology platform, the Gilles Thomas Bioinformatics Platform, the biosamples management platform, the clinical staff and the molecular tumour board. The molecular tumour board is made up of medical oncologist, pathologist, molecular biologists, bioinformaticians and data scientists meeting every week to recommend matched molecular-targeted agents including immunotherapies and including those accessible in clinical trials [6].

The ProfiLER01 program enrolled 5,991 patients between February 2013 and November 2023 and were ongoing at this time. On the basis of these data, our team has previously described the molecular characteristics of several population including patients with gastro-oesophageal cancers [7], patients with alterations in homologous recombination-related genes and distinct platinum response in metastatic triple-negative breast cancers [8], patients with primary brain tumours [9], refractory gynaecological cancers [10], metastatic sarcomas [11] and paediatric tumours [12].

### Other molecular analysis programs

The ‘France Génomique plan 2025’ (FMG2025) provides whole genome sequencing (WES) and RNAseq for patients with refractory diseases. Analyses are performed on two platforms: Auvergne Rhônes Alpes Genomique (AURAGEN) in Lyon covering the analysis of Southern France and Sequencing Omics Information Analysis (SeqOIA) in Paris covering the analysis of Northern France [13]. It proposes extensive molecular testing with WES and RNA sequencing for multiple diseases including 60 types of rare diseases, uncharacterized suspected genetic predispositions and eight indications in oncology: refractory cancers, rare cancers, cancer of unknown primary and haematology. The first patients were included in October 2019 and up to early 2023, 8,447 reports were generated including 1,969 patients with cancer.

Another program, PRISM-Portal, evaluates the impact of ctDNA at the start of metastatic disease, during treatment and/or at progression. The proportion of patients with ctDNA sequencing has helped guide therapy.

The PLANET program (NCT05099068) aims to generate sequential molecular analysis for patients treated with standard therapies, including detection of mutations, amplifications, insertions/deletions, microsatellite instability, mutational burden and expression alteration using RNA Sequencing either on tumor and/or liquid biopsies.

## Genomic-driven clinical trials

### MOST-MOST Plus and MEGAMOST

MOST-MOST Plus and MegaMOST trials are composed of multiple treatment cohorts defined by the combination of a targeted treatment and a biomarker derived from molecular profiling. New cohorts are opened on a regular basis through the integration of new study treatments, generally in indications unexplored by pivotal pharma-initiated trials. Both have adaptive Bayesian approach, futility interim analysis and a target of 50 patients analysed for the primary endpoint for each cohort [14]. A Bayesian approach allows updating knowledge gradually rather than restricting revisions in a trial design with fixed sample sizes. The main selection criteria include adult patients with metastatic or unresectable solid tumours of any type, not amenable to curative treatment, and those who previously received at least one prior systemic treatment regimen.

### MOST

**Figure 1:**
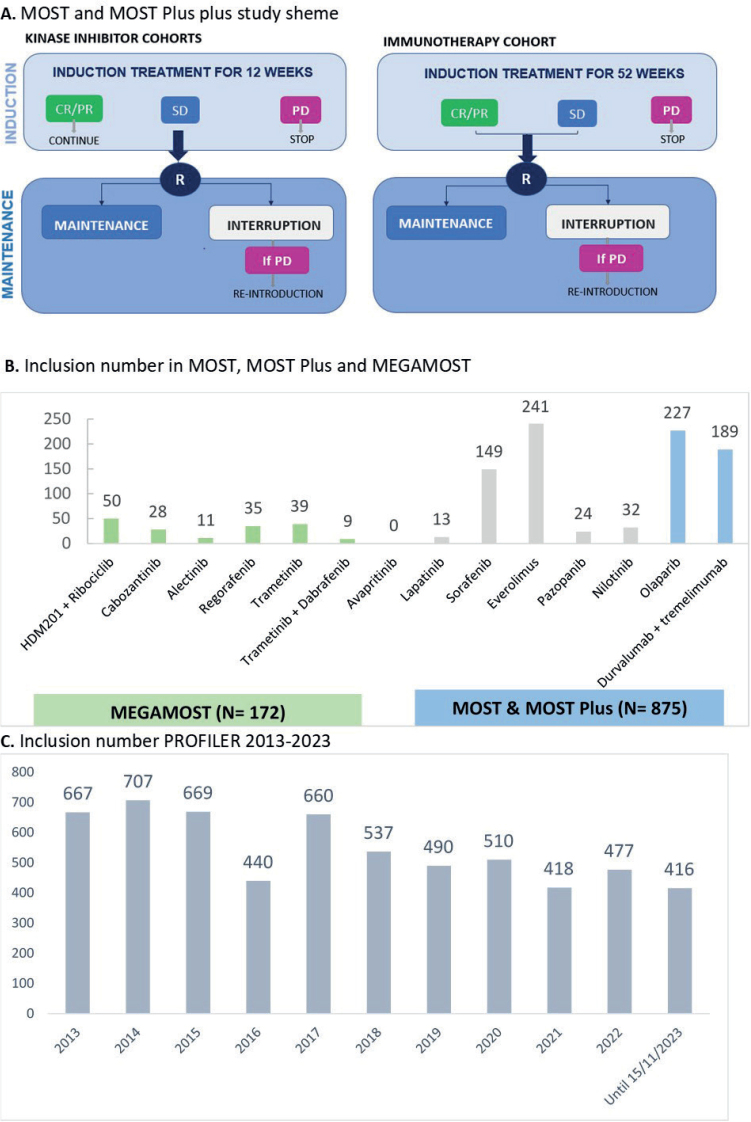
A. the MOST plus study scheme and B. Inclusions in MOST, MOST Plus and MEGAMOST In blue (MOST and MOST-Plus) or green (MegaMOST): ongoing cohorts, in grey: cohorts closed. Of note, avapritinib cohort was opened in October 2023. C. Inclusion number PROFILER 2013–2023. MTT: Molecular Target Therapy; CR: complete response; PR: partial response; SD: stable disease; PD: progression disease; R: randomisation.

The MOST program (NCT02029001) started in 2014 with a multiarm, genomic-driven Phase II trial, conducted using a randomised discontinuation design. This is a way to evaluate the efficacy of molecular targeted agent oriented towards a matched molecular alteration in a randomized fashion. After an induction period of treatment of 12 weeks, patients with stable disease are randomly assigned (1:1) to continuation or interruption of matched therapy defining the maintenance period (Figure 1). Between 2014 and November 2023, we enrolled 427 patients in five cohorts with the molecular targeted agents lapatinib, sorafenib, everolimus, pazopanib, or nilotinib oriented by predefined somatic alterations (Table 2). The trial is running in six French sites (Centre Léon Bérard, Hospices Civil de Lyon, Institut Curie, Institut Paoli Calmettes, Oncopole Toulouse, Institut Bergonié). The primary endpoint is progression-free rate at 16 weeks after randomisation.

**Table 2:** Cohorts of the MOST+ and MEGAMOST trial.

Molecular alterations*	Study drug’s name and Dosage regimen	Eligible histological tumour types	Partner
Documented amplification of CDK6 and/or CDK4 and/or CDKN2A homozygous deletion and/or amplification of CCND1 and/or CCND3 with no deletion/losses more than single copy of RB1 by copy number and P53 wild-type	**ribociclib** 200 mg/day, QD, 2 weeks on/1 week off, PO + **HDM201** 120 mg, Q3W, PO	Adult any solid tumours (excluding gliomas)	Novartis
AXL, MET, VEGFR, VEGF, RET, ROS1, MER, TRKB, TIE-2 and/or Tyro3 activating mutation and/or amplification and/or NTRK translocation	**cabozantinib**, 60 mg/day, continuous, PO	Adult with any solid tumour	Ipsen
Activating ALK alterations: translocation or selected mutations (for instance R1275Q, F1245C, F1174X or listed Appendix 9)	**alectinib**, 600 mg BID, PO	Adult patients with advanced or metastatic • Cohort 1: Inflammatory myofibroblastic tumors • Cohort 2: Neuroblastoma, • Cohort 3: other tumours with ALK alterations.	Roche
Activating mutation and/or amplification of VEGFR1-3, TIE-2, KIT, RET, RAF1, BRAF (other than V600 mutations), CRAF, HRAS, KRAS, PDGFR, FGFR1-2, FLT3 and/or CSF1R and/or amplification of the ligands and/or biallelic inactivation of SMAD4	**regorafenib** 160 mg, once daily, 3 weeks on/1 week off, PO	Adult with any solid tumours	Bayer
Activating mutation and/or amplification of KRAS (except KRAS G12C), NRAS, HRAS and/or MAP2K and/or biallelic inactivation ^₿^ of NF1 and/or activating mutation PTPN11 and/or amplification or translocation of BRAF	**trametinib** 2 mg/day, continuous, PO	Adult with solid tumour, excluding melanoma Lung cancers with KRAS G12C mutation, CRC and PDAC with KRAS mutations.	Novartis
BRAF V600 mutation	**trametinib** 2 mg/day, continuous, PO + **dabrafenib** 150 mg BID, PO	Adult with solid tumor, excluding Melanoma, Lung cancers, CRC.	Novartis
Activating mutations of KIT exon 17 or PDGFRA exon 18 associated or not to mutation on KIT exon 11 or PDGFRA exon 12/14	**avapritinib** 300 mg/day, continuous, PO	Adult with any solid tumour	Blue Print
Mutations of ABL1, KIT, PDGFRA, PDGFRB, DDR1, DDR2, CSF1R, or amplification/translocation of the genes and/or ligands	**nilotinib** 400 mg/day continuous, PO	Only pigmented villonodular synovitis, not amenable to curative treatment	Novartis
Mutations or amplification of the PIK3CA, PIK3R1, AKT1, AKT2, mTOR, RICTOR, RAPTOR genes or with TSC1, TSC2 or PTEN loss (defined as a complete loss of both gene copies OR loss of one copy + mutation on the other copy OR loss of one copy + loss of expression using IHC)	**everolimus** 10 mg mg/day continuous, PO	Adult with any solid tumour	Novartis
Mutations of VEGFR1-3, PDGFRA, PDGFRB or KIT or amplification/translocation of the genes and/or of the ligands	**pazopanib** 800 mg/day continuous, PO	Adult with any solid tumour	Novartis
Mutations of VEGFR1-3, PDGFRB, FLT3, BRAF (other than V600 mutations), CRAF, HRAS, KRAS or RET or amplification/translocation of the genes and/or of the ligands	**sorafenib** 400 mg BID continuous, PO	Adult with any solid tumour	Bayer
Mutations or amplifications of HER2	**lapatinib** 1,500 mg/day continuous, PO	Adult with any solid tumour	Novartis
Mutation only if double hit documented: ATM, BAP1 et BRIP1 Mutation : BRCA2, BRCA1, RAD51C, PALB2, RAD51D Loss: BRCA1, BRCA2, ATM and BAP1 Mutation + heterozygote deletion: BRCA1, BRCA2, ATM and BAP 1	**olaparib** 300 mg BID	Adult with any solid tumour except in prostate, or stomach cancers and except for patients eliglible to olaparib’s available labels and reimbursements in France	Astra Zeneca
Tumour mutation burden >10 Muts/Mb on liquid biopsy MSI-High PD1/PDL1/CTLA4 amplification MLH1/MSH2/MSH6/PMS2 POLD1 & POLE mutation or LOH except lung or urothelial or head and neck tumours	**durvalumab** 1,500 mg/day, Q4W, PO + **tremelimumab** 75 mg/day, Q4W, PO	Adult with any solid tumour	Astra Zeneca

The MOST sorafenib cohort was composed of 151 patients with at least one of the following molecular alterations: mutations or amplification/translocation in VEGFR1-3, PDGFRB, FLT3, BRAF (excluding V600E), CRAF, HRAS, KRAS, or RET, and/or cognate ligands. For the induction period, 35 patients had SD at 12 weeks. The progression-free rate at 16 weeks after randomisation was 65% [95%CI 43.4–83.7] in the continuation arm, with a significant increase in PFS (5.6 months [95%CI 1.97–6.77] versus 2.0 months [95%CI 1.61–3.91], *p*-value = 0.0231). The progression-free rate in the interruption arm was 25% [7.8–48.1]. The median survival was also improved from 4.3 [95%CI 8.9-23.8] in the interruption arm to 8.0 months [95%CI 3.5-15.2] in the continuation arm, *p*-value = 0.0857. It suggests that sorafenib matched to molecular alterations improved the outcome of patients with SD compared with its interruption. Grade 3 or higher sorafenib-related adverse events were reported in 67 patients (46.2%), as hypertension, vomiting, fatigue, hand and foot syndrome [15].

The nilotinib cohort continues only for advanced pigmented villonodular synovitis (TGCT/PVNS), a group of locally aggressive tumours with activation of the CSF1R pathway [16]. The everolimus, pazopanib and lapatinib cohorts are closed to enrolment and under analysis. Although there is a randomisation for comparative analysis, a potential limitation in the interpretation of the results of the MOST trial is that it does not include a control group of patients not driven on prespecified genomic alterations.

### MOST Plus

MOST Plus is an amended version of MOST (NCT02029001) with the addition of 2 cohorts of patients treated with olaparib or the combination of durvalumab and tremelimumab (Table 2). The induction period of treatment is 12 weeks for olaparib and 52 weeks for immunotherapy before randomisation of patients with stable disease (for olaparib) or stable disease and objective response (for D+T cohort). The MOST Plus durvalumab and tremelimumab is ongoing and recruited 189 patients up to November 2023. The MOST Plus olaparib cohort, presented at ESMO2023, included 213 patients with somatic or germline mutations in homologous recombination genes such as BRCA1/2, RAD51, PALB2, ATM, etc. (beyond current label in oncology). Among the 213 patients who received olaparib (300 mg, BID), 6% (*n* = 14) had partial response at 12 weeks and 16% had stable disease, with a 3-month PFS rate of 23% (48/213). For patients with partial responses, 8 had breast cancer, 3 pancreatic cancers, and the 3 remaining had prostate, uterine or bladder cancers, most of them harbouring biallelic alterations in homologous recombination genes. Among all patients, 23.6% with PALB2 mutations had partial responses. Grade 3 or higher adverse events were reported in 81 patients (38%) and 14.1% of patients discontinued treatment due to adverse events [17]. Based on this analysis, we recently updated the molecular selection criteria for future patients enrolled in this cohort: alterations on the genes BRCA2, BRCA1, RAD51C, RAD51D, PALB2, BAP1, ATM and BRIP1.

### MEGAMOST

MEGAMOST (NCT04116541) is an ongoing phase II, genomic-driven adaptive Master protocol. Patients are assigned to a treatment cohort based on molecular alterations/characteristics detected on tumour samples (from primary tumour or metastatic lesion) or liquid biopsy. MEGAMOST is currently running in six French sites (Centre Léon Bérard, Centre Antoine Lacassagne, Institut Bergonié, Institut Paoli Calmettes, Oncopole Toulouse and Gustave Roussy Cancer Center). Up to November 2023, 172 patients were enrolled in seven cohorts of molecular targeted agents (HDM201 and ribociclib, alectinib, regorafenib, trametinib, trametinib and dabrafenib, avapritinib) in advanced/metastatic solid tumours. The primary objective is to evaluate the activity of selected study drugs for each cohort based on molecular alterations characteristics of the patient’s tumour (progression free rate after 3 months of treatment). A Bayesian statistical approach is regularly analysing the efficacy of each cohort. The patients’ recruitment is ongoing in each cohort and no publication is already available.

## Conclusion and perspectives

**Figure 2:**
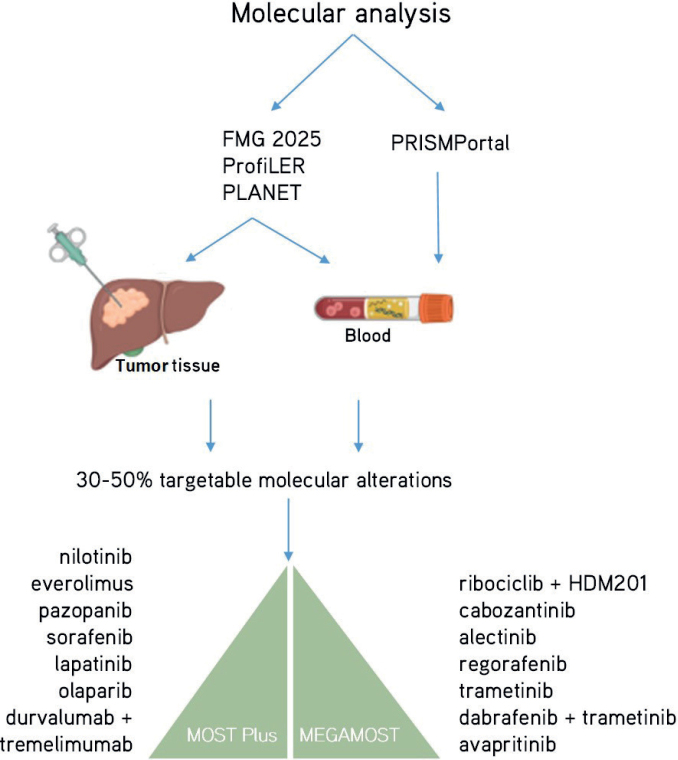
The molecular diagnostic programs are used to orient patients to the MOST Plus and MEGAMOST clinical trials.

The high failure rate of clinical development in oncology is mainly due to the erroneous hypothesis that all patients affected by a similar tumour type would be biologically identical (this is represented by selection criteria of clinical trials oriented on tumour types). The MOST trials are clearly aiming at repositioning molecular targeted agents with a personalized medicine strategy (Figure 2). The success of repurposing molecular targeted agents in oncology is supported by the recent analysis of the main factors leading to the best Likelihood of FDA Approval (LoA) for pharmaceutical compounds together with their companion diagnostic tools, namely (1) rare disease therapy (LoA = 17%), (2) development of a treatment with biomarkers (i.e. companion diagnostic tools, LoA = 16%), and (3) prior approval (i.e. repositioning, LoA +3.6%) [18, 19]. When a cohort meets the efficacy endpoint in a cohort of a DRUP-like trial, it can support drug approval and reimbursement in the participating country. For example, nivolumab, an immune-checkpoint inhibitor targeting anti-PD1, obtained approval and reimbursement in the Netherlands on July 1st, 2022, based on a cohort of the DRUP trial evaluating the treatment of dMMR/MSI solid tumours of any origin [5, 20, 21]. Nevertheless, two teams in the PCM4EU consortium showed that up to 40–50% of patients with rare cancers could have a genomic-driven orientation if treatments were available and accessible in the country of the patients [4, 5]. To this end, DRUP-like trials such as MOST trials include a process of public, open, and shared evaluation of the treatment efficacy. The collaboration of several DRUP-like trials on data sharing will support an efficient process to approve compounds repurposing in rare cancers.

## Data Availability

No data used in this study, only review of the literature unless mentioned
